# Revisiting the genotoxic syndrome: Why are we overlooking the ecological impacts of genotoxins on animal populations?

**DOI:** 10.1590/1678-4685-GMB-2025-0055

**Published:** 2025-09-15

**Authors:** Joel Henrique Ellwanger, Marina Ziliotto, José Artur Bogo Chies

**Affiliations:** 1 Universidade Federal do Rio Grande do Sul (UFRGS), Departamento de Genética, Programa de Pós-Graduação em Genética e Biologia Molecular, Laboratório de Imunobiologia e Imunogenética, Porto Alegre, RS, Brazil

**Keywords:** Biodiversity, ecology, genotoxicity, pollution, wildlife

## Abstract

Cancer, neurodegeneration and other chronic diseases have been a primary focus for researchers studying the effects of genotoxins on human populations. However, when the scope shifts to non-human animals in natural environments, the impact of genotoxins extends beyond aging-related diseases. In ecological studies, DNA damage biomarkers (e.g., fragmentation of genetic material, micronuclei) in animal populations are often used to indicate “environmental quality”, but usually with a focus on human health. For example, an increase in the micronuclei frequency in an animal (sentinel) population may indicate risks to the genomic stability of the human population living in the same environment. This human-oriented approach frequently overlooks the ecological impacts of genotoxins on animal populations as an end, limiting the benefits for nature conservation from geno-toxicological studies. Environmental genotoxins pose a significant threat to biodiversity by causing multiple classes of DNA damage and mutations that impair varied cellular functions and reproduction, ultimately jeopardizing species survival through non-carcinogenic processes. This phenomenon, termed “genotoxic syndrome”, will be revisited in this article with examples of its detrimental effects on non-human animal populations. Finally, challenges and opportunities in evaluating genotoxic syndrome and its importance for conservation strategies are also discussed.

## Introduction: a brief update on genotoxins and their significance in ecology

Genotoxic toxins, or “genotoxins” are physical (e.g., radiation) or chemical agents capable of causing mutations and other types of DNA alterations (e.g., inversions, deletions, additions, adducts, single- or double-strand breaks, micronuclei and other types of chromosome aberrations) on somatic and germ cells, in all types of living organisms (Würgler and Kramers, 1992; Medina et al., 2007). Exposure to genotoxins results in deleterious consequences when cellular detoxification and DNA repair systems fail or are insufficient to deal with the genetic insult (Depledge, 1998). When this occurs, genotoxins can (I) affect DNA structure or organization and (II) influence gene expression through DNA methylation, histone modification, altered expression of non-coding RNA, among other mechanisms. Also, downstream of these direct modifications to genome structure and regulation, genotoxins can (III) modify allele frequencies due to selective pressures, as well as (IV) impact genetic diversity and reduce fitness (Anderson et al., 1994; Depledge, 1998; Chappell et al., 2016; Medina *et al.*, 2007). Notably, aspects concerning genetic diversity and fitness will be discussed in more detail later.

Currently, chemical pollution is a major planetary challenge, posing a significant threat for human health and biodiversity from varied ecosystems (Fuller et al., 2022; Groh et al., 2022; Sigmund et al., 2023). Several chemical pollutants present in the environment exhibit genotoxic activity, including toxic metals (Zocche et al., 2013; Dourado et al., 2017; Matos et al., 2017), pesticides (Bolognesi, 2003; Zeyad et al., 2019), medicinal drugs (Parrella et al., 2015), and microplastics (Menezes et al., 2024). Genotoxins are usually studied in the context of human health, as they influence processes related to carcinogenesis (Roos et al., 2016) and aging (Yousefzadeh et al., 2021), significantly affecting the risk of neurodegeneration (Kisby et al., 2024) and several non-communicable diseases (Milic et al., 2015). However, although an overlooked issue, the study of genotoxins has an important meaning also for ecology and nature conservation.

The goal of genetic ecotoxicology is “*to assess, predict, and prevent significant radiation- or chemical-induced genetic and epigenetic damage in populations*” (Anderson et al., 1994). In ecotoxicology, genotoxicity markers (e.g., DNA damage, micronuclei) in cells of natural biota are usually employed to measure, or indicate levels of, “environmental quality” (Anderson *et al.*, 1994; Theodorakis, 2001). Moreover, even within the field of ecotoxicology, these genetic biomarkers are classically used to indicate risks to human health, often employing the animal only as an environmental “indicator of exposure” (Shugart, 2000; Depledge, 1994). Even though correct, this approach, as previously mentioned, often overlooks the impacts of genotoxic agents on animal health as an end, disregarding the harmful effects of genotoxins on animals, as well as the subsequent consequences on biodiversity and stability of ecosystems.

Although cancer is relatively common across various animal species (Albuquerque et al., 2018; Vincze et al., 2022) and can act as a shaping-force in ecological networks (Perret et al., 2020), its health burden in non-human animal populations is different from that observed in humans. In natural environments, many animals die before developing cancer (or before the disease progresses to advanced stages) due to natural factors such as predation or competition, as well as due to anthropogenic pressures (e.g., hunting, land-use changes, habitat loss). Additionally, some species, such as invertebrates, may exhibit natural resistance to environmental agents that induce malignant diseases, and genotoxic effects in these populations often lead to outcomes unrelated to cancer. As a result, cancer may not always be the most relevant outcome to prioritize in ecotoxicological studies (Depledge, 1994, 1998; Perret *et al.*, 2020). Therefore, studies on the impact of genotoxins on animal populations should adopt a focus distinct from that employed in human health studies.

## Consequences of genotoxicity in animal populations

### Evolutionary effects

The consequences of genotoxins in animal populations go far beyond cancer. Genotoxic pollutants affect a wide range of cells and biological systems, contributing to biodiversity loss and impacting the course of evolution (Medina et al., 2007; Johnson et al., 2024; Ziliotto et al., 2024a). In sexual organisms, genetic variability arises not only through meiotic recombination but also through spontaneous mutations, chromosomal rearrangements, transposon activity, mitotic recombination, and gene amplifications. These mechanisms, while inherently random, can also be triggered or amplified by environmental agents, such as pollutants with genotoxic activity (Würgler and Kramers, 1992). Furthermore, in addition to activating genotoxicity mechanisms, exposure to pollutants also affects hormonal balance, metabolic pathways involved in toxicant metabolism, and other biological processes. Consequently, genotoxicity and the direct effects of chemical toxicity occur simultaneously, impacting both individuals and populations (Depledge, 1994, 1998; Ziliotto *et al.*, 2024a).

Exposure to genotoxins can increase mutation rates (Theodorakis, 2001; Johnson et al., 2024), being a major driver of changes in community structure and population fitness, as well as micro-evolution (Medina et al., 2007). Of note, micro-evolution due to pollution characterizes rapid genetic changes or small-scale evolutionary processes directed by environmental exposure to toxic pollutants (Medina *et al.*, 2007). Variability caused by mutations in germ cells allows species to adapt to new and changing environments, with genetic variability (translated phenotypically) being fundamental to the evolutionary process. In other words, in certain instances, new variability, spontaneously or induced by environmental genotoxins, can favor the adaptation of species to new environments or to the influence of already existent selective pressures (Würgler and Kramers, 1992). In polluted environments, the selection of critical alleles for survival may occur (Bickham and Smolen, 1994), often through the elimination of sensitive genotypes to genotoxic exposure (Anderson et al., 1994). However, this process can lead to changes in gene pools and a reduction in the genetic variability of exposed populations, a phenomenon known as “genetic erosion” (Van Straalen and Timmermans, 2002). For example, insecticide resistance alleles can persist in insect populations for generations, even after pesticide use has ceased. As generations pass, reduced genetic variability can lead to several adverse outcomes, including decreased population abundance, increased susceptibility to diseases, reduced reproductive success and, in extreme cases, population extinction (Anderson *et al.*, 1994; Theodorakis, 2001). Genotoxic pollutants can also cause a bottleneck effect, reducing population size (Van Straalen and Timmermans, 2002; Mussali-Galante et al., 2014).

While we have briefly discussed how variability caused by mutations in germ cells enables species to adapt to new environments, it is essential to emphasize that excess of mutations in germ cells can be harmful, frequently resulting in fitness loss. Indeed, most mutations are deleterious or neutral, rarely conferring any selective advantage on the organism (Würgler and Kramers, 1992; Johnson et al., 2024). Notably, genotoxic pollutants can influence the adaptive process by causing mutations and other types of genetic alterations (e.g., cumulative DNA damage) in germ cells that reduce fertility (e.g., germ cell death due to apoptosis) and even induce embryonic mortality due to lethal mutations or significant genetic damage (Würgler and Kramers, 1992; Anderson and Wild, 1994; Theodorakis, 2001). The limited DNA repair capacity observed in gametes can explain, at least partially, these outcomes observed in invertebrate and vertebrate animals (Anderson and Wild, 1994). Furthermore, non-lethal mutations in germ cells can introduce new deleterious genetic characteristics into the gene pool of a given species, affecting other traits important for the survival of individuals at reproductive age (Würgler and Kramers, 1992). 

In other words, genetic variability is essential for the survival of species. Exposure to genotoxins imposes strong selective pressure, potentially benefiting populations with high mutation rates by enhancing adaptation and survival. However, the gain in genetic variability comes at a cost to a portion of the population. Individuals carrying neutral or advantageous mutations may persist while individuals carrying harmful mutations may be eliminated from the population (Würgler and Kramers, 1992; Johnson et al., 2024). Although mutations are essential for adaptation to new environments, an abnormally high rate of deleterious mutations, such as those induced by excessive environmental pollutants, can endanger a population and represent an issue to biodiversity conservation. Consequently, this can disrupt the balance of food webs and alter population size and structure, ultimately impacting the ecosystem where the species lives (Würgler and Kramers, 1992; Anderson and Wild, 1994; Anderson *et al.*, 1994; Depledge, 1998; Medina et al., 2007).

In somatic cells, DNA damage and other genetic alterations will become a dead ending, not passing on to subsequent generations. However, this does not mean that exposure to genotoxins does not have important consequences for such cells and their respective organisms. Excessive DNA damage and genetic alterations in somatic cells can impair cellular and enzyme functions, weaken immune responses, alter metabolism and nutrient processing, hinder toxin elimination, and contribute to the development of diseases. All these processes affect the survival and well-being of species in nature (Kurelec, 1993; Depledge, 1994; Ziliotto et al., 2024a). These effects, especially when observed in animals of reproductive age, can impact health and fitness and significantly diminish reproductive success, compromising long-term population growth rates and viability (Depledge, 1998; Johnson et al., 2024). As previously discussed, changes in traits that affect the reproduction and fertility of species have the potential to modify the survival and adaptation of species in natural environments. Furthermore, pollutant exposure can lead to population bottlenecks and inbreeding, which may increase the likelihood of extinction. In summary, genotoxic pollutants modify the evolutionary process by affecting both germ and somatic cells since genotoxins can directly affect DNA or act as selective pressures for genetic changes (Würgler and Kramers, 1992; Bickham and Smolen, 1994).

### Genotoxic syndrome

Dealing with genotoxins and DNA damage has a significant cost for cells due to the recruitment of enzymes and mechanisms of detoxification and DNA repair. Increased enzyme (protein) turnover required to cope with genotoxin-induced DNA damage will have important consequences on metabolic expenditure and fitness (Kurelec, 1993; Depledge, 1994). The cellular, reproductive, physiological, metabolic and ecological impacts of genotoxins on non-human populations living in natural environments were named “genotoxic disease syndrome” in the 1990s (Kurelec, 1993) by the Macedonian ecotoxicologist Branko Kurelec (1935-1999) (Peakall and Shugart, 2000). While this expression captures the effects of genotoxins on natural populations, we propose simplifying it to “genotoxic syndrome”, as many of the consequences of genotoxic exposure do not fit the classical definition of disease, with visible signals and symptoms.

In this context, this article aims to revisit the effects of genotoxic syndrome on animals, discussing how environmental genotoxins can impact multiple biological parameters in natural populations as downstream consequences of genomic damage. Understanding these effects is crucial for gaining deeper insights into how biodiversity declines and how environmental pollutants influence the course of evolution. Finally, we also discuss some challenges and opportunities for the evaluation of genotoxic syndrome and its significance for strategies of nature conservation.

## Revisiting the evidence of genotoxic syndrome

Table 1 highlights studies that evidence the diverse effects of genotoxins on animal species, ranging from molecular damage to reproductive issues. The studies cited in Table 1 and throughout this narrative review were selected from a non-systematic search conducted in 2024 and 2025 using search terms (alone or combined) related to genotoxicity and ecology (e.g., “animal”, “DNA damage”, “environment”, “evolution”, “fitness”, “genotoxic”, “genotoxic syndrome”, “genotoxicity”, “pollution”, “reproduction”), regardless of publication year. The search was conducted utilizing the search tools Google Scholar (Google, 2025) and PubMed (NIH, 2025). Sources cited in the reference lists of the selected articles were also considered for this review, as well as articles from the authors’ personal libraries. Table 1 aims to provide selected examples of genotoxic syndrome, rather than an exhaustive list, which is the same proposal of this narrative review. Also, we emphasize that Table 1 compiles studies that provide integrated evidence of genotoxic syndrome (biological issues as consequences of genomic damage) in natural and experimental settings, rather than merely reporting genotoxicity data.

**Table 1 -:** Examples of genotoxic syndrome-related consequences in various animal species.

Genotoxin	Species	Main outcomes	Reference
Radiation	Polychaete worm (*Neanthes arenaceodentata*)	Genotoxicity-related detrimental reproductive effects; Induction of dose-dependent chromosomal aberration; Increased mortality and reduction of lifespan only in high doses.	Anderson et al. (1990)
Benzo[*a*]pyrene	Frog (*Xenopus laevis*)	Increased levels of DNA adducts and micronuclei induced by dose-dependent exposure were associated with short- and long-term effects on larval growth and development. The data suggested these effects as indicators of sublethal genotoxic exposure and DNA damage.	Sadinski et al. (1995)
Traffic pollution	Wild rodents (*Mus domesticus*)	Increased frequency of micronucleated erythrocytes and abnormal sperm cells.	Ieradi et al. (1996)
Radiation	Barn swallows (*Hirundo rustica*)	Increased germline mutations and fitness loss.	Ellegren et al. (1997)
Oil	American mink (*Mustela vison*) and sea otter (*Enhydra lutris*)	Somatic chromosomal damage and possibly heritable alterations of genome size.	Bickham et al. (1998)
Mercury, polychlorinated biphenyls, and other compounds	Redbreast sunfish (*Lepomis auritis*)	Higher biomarker response and mutagenicity on more polluted points; Community diversity levels were inversely proportional to the pollution level of the evaluated area, whereas pollution-tolerant species levels were higher on more polluted points.	Theodorakis et al. (2000)
4-nitroquinoline-1-oxide	Zebrafish (*Danio rerio*)	Genotoxicity-related reduction in egg production and increased extinction risk.	Diekmann et al. (2004)
Tritiated water	Mollusk (*Mytilus edulis*)	Even low doses of β-radiation emitted by tritiated water can induce genetic damage in the embryo-larval stages of marine mussels and potentially accumulate at cellular and individual levels, causing cytogenetic damage, cytotoxicity, developmental abnormalities, and mortality in a dose-dependent level.	Hagger et al. (2005)
Tributyltin	Dog-whelk (*Nucella lapillus*)	DNA damage was positively correlated with the degree of imposex in animals that exhibited tributyltin-induced imposex. Tributyltin body burden was also associated with prevalence of abnormal growths.	Hagger et al. (2006)
Ionizing radiation	Zebrafish (*Danio rerio*)	Genotoxic stress caused by ionizing radiation exposure induced senescence-associated characteristics (i.e., impaired fin regeneration, decline in reproductive abilities, increased senescence-associated β-galactosidase activity, and a reduction in melatonin secretion).	Tsai et al. (2007)
Benzo[*a*]pyrene	Crustacean (*Daphnia magna*)	Decrease fitness, in number of eggs, maximum body size and number of broods at a dose-dependent level.	Atienzar et al. (1999)
Methyl methanesulfonate, potassium dichromate	Crustacean (*Gammarus fossarum*)	Higher sensitivity of spermatozoa to DNA damage caused by genotoxins compared to oocytes, probably due to its inability to repair DNA. A link between DNA damage in sperm and the abnormalities in embryos was observed.	Lacaze et al. (2011)
Ethyl methane sulfonate	Shrimp (*Artemia franciscana* and *Artemia parthenogenetica*)	DNA damage positively correlated to negative effects on individual and population fitness of both sexual and asexual species. Comparisons between sexual and asexual reproduction showed that sexual reproduction is more effective in combating the multigenerational effects of genotoxicity.	Sukumaran and Grant (2013, b)
Cadmium	Caterpillar (*Lymantria dispar*)	Increase in DNA damage, reduction on hemocyte viability and larval mass, prolongation of the fourth instar and total development time in a dose-dependent manner.	Matić et al. (2016)
Industrial sewage sludge	Mollusk (*Biomphalaria glabrata*)	Nuclear abnormalities, apoptosis, and nucleoids with a high degree of fragmentation in their structure.	Siqueira et al. (2020)
Aluminum chloride	Swiss mice (*Mus musculus*)	Genotoxicity and DNA damage in all tested doses. *In vivo* testing revealed increased numbers of micronuclei formation in all concentrations and indications of systemic toxicity.	Paz et al. (2017)
Gamma radiation	Zebrafish (*Danio rerio*)	Persistent genotoxicity and reproduction impairment.	Hurem et al. (2018)
Uranium	Crustacean (*Daphnia magna*)	Genotoxic effect; Loss of DNA integrity and short-duration transmission of DNA damage and strand breaks to offspring.	Reis et al. (2018)
Oxyfluorfen (herbicide)	Mollusk (*Biomphalaria glabrata*)	DNA damage in acute and chronic exposure, nuclear abnormalities and apoptosis.	de Vasconcelos Lima et al. (2019)
Metals and pesticides	Frog (*Rhinella fernandezae*)	Worst body condition and high micronuclei frequency in animals collected in the agriculture-surrounded site that presented lowest environmental quality of the analyzed sites.	Peluso et al. (2020)
Emissions from unsanitary landfill	Wild black rats (*Rattus rattus*)	Micronuclei formation, abnormal sperm morphology in cauda epididymis (biomarkers of genotoxicity) and changes in hematological indices, erythrocyte morphology, and pathological lesions of the various tissues (biomarkers of systemic toxicity).	Alimba et al. (2021)
Glyphosate-based herbicide	Tadpoles (*Physalaemus cuvieri* and *Physalaemus gracilis*)	Micronuclei formation and several types of erythrocyte nuclear abnormalities in all tested concentrations.	Herek et al. (2021)
Pesticides	Frog (*Leptodactylus luctator*)	Pesticide bioaccumulation in liver and nuclear abnormalities, with potential impacts on population decline.	Ascoli-Morrete et al. (2022)
Radiation	Root voles (*Microtus oeconomus*)	Adaptive rearrangements in chronically irradiated animals; Genome instability in offspring.	Raskosha et al. (2023)

**Figure 1 -:**
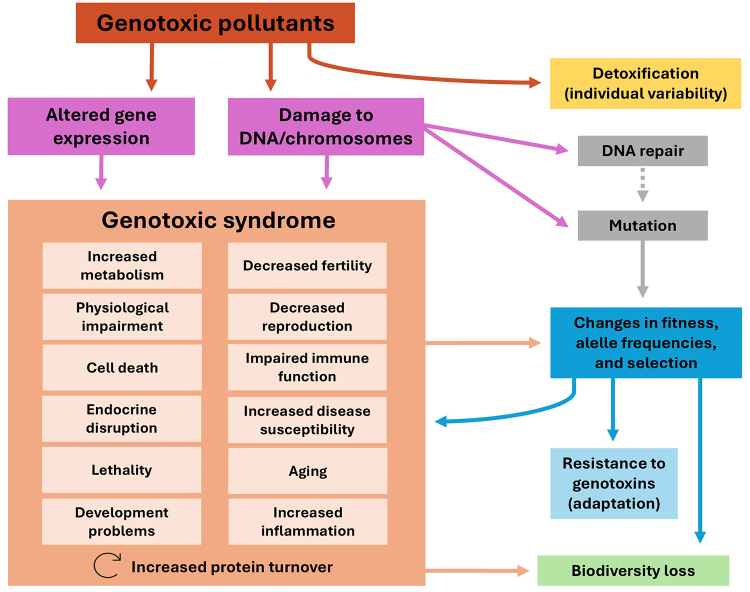
Consequences of genotoxic syndrome resulting from exposure to genotoxic pollutants. This figure provides a summary of how genotoxic pollutants cause genotoxic syndrome in general. Of note, each species has particularities in terms of exposure to pollutants, detoxification mechanisms, susceptibility to genetic damage, and DNA repair. These characteristics can affect the processes summarized in the figure and should be taken into account in studies evaluating a particular species.

As evidenced by our review, genotoxic damage is usually accompanied by other types of cellular and tissue damage (Table 1). Indeed, environmental genotoxins often cause DNA alteration along with other cellular changes, such as protein thiol oxidation reactions and dysfunctional cellular metabolism (Depledge, 1998). Changes at the molecular, cellular or tissue level can have important effects on the ecological macroscale. For instance, nuclear abnormalities can induce teratogenesis and decreased species fitness (Peluso et al., 2020). This and the other examples cited in Table 1 confirm that genotoxic syndrome has important, but overlooked, impacts on several animal populations belonging to different taxonomic groups. Figure 1 summarizes the multiple consequences of the genotoxic syndrome caused by exposure to genotoxic pollutants.

## Challenges and opportunities for evaluation of genotoxic syndrome

Although all species are susceptible to genotoxic syndrome, susceptibility to the effects of pollutants can vary both between different species and within individuals of the same species. In a given ecological community, some individuals are more resistant to genotoxic effects than others due to biological characteristics (e.g., presence or absence of chitin, more or less robust detoxification systems), behavioral characteristics (e.g., searching for food in highly polluted places, such as landfills), type of habitat (e.g., urban animals may be more exposed to genotoxins than rural animals), as well as natural and anthropogenic characteristics of each habitat, such as the presence of toxic metals due to volcanic eruptions or mining activities (Depledge, 1994; 1998; Ceyca-Contreras et al., 2020; Ellwanger et al., 2025a). For example, species that live in direct contact with the soil (e.g., rodents, invertebrates) are exposed to a greater load of soil-associated pollutants (Ellwanger *et al.*, 2025a). Also, in amphibians, exposure to genotoxic pollutants varies depending on the specific ecological habits and behaviors (e.g., egg deposition site) of each species (Gonçalves et al., 2019).

Moreover, each species exhibits specific susceptibilities in relation to the capacity of the genetic material to interact with genotoxic agents, influencing parameters such as genomic stability and the regulation of gene expression (Ellwanger et al., 2025b). Also, although many proteins involved in DNA repair are conserved across different species, the capacity and characteristics of DNA repair systems varies across taxonomic groups, and even between individuals of the same species, thereby affecting mutation rates and other genotoxicity-related factors, including disease incidence and fitness (Depledge, 1994; Eisen and Hanawalt, 1999; Nischwitz et al., 2023). For example, DNA repair genes are upregulated in longer-lived naked mole rats compared with short-lived mouse (MacRae et al., 2015).

These variabilities will have consequences for the population and can interfere with genetic ecotoxicological assessments (Depledge, 1994), possibly including interspecies ecological relationships, which are more difficult to measure *in situ*. When analyzing genotoxicity biomarkers in natural populations living in extremely polluted environments, only the living individuals that are most resilient/adapted to the contaminated environment are usually sampled. The other individuals that are more sensitive to pollutants have died or migrated to less polluted locations (Bickham and Smolen, 1994).

In evolutionary terms, exposure of animals to anthropogenic pollution is a new event and therefore chemical agents are a significant selective pressure. Organisms resistant to pesticides (e.g., insects) and antibiotics (e.g., bacteria) emerge rapidly from populations exposed to these chemical agents, being good examples of rapid micro-evolution (Brown et al., 2009). The potential consequences of micro-evolution due to pollution are diverse and can affect the composition of communities and even the equilibrium of ecosystems (for a more complete overview of these topics, see Medina et al., 2007). Many of these consequences, although biologically plausible and predicted in theory, may be difficult to observe in the field or in laboratory studies.

In research addressing ecotoxicological genetics, various economic, logistical, and ecological limitations hinder the ability to sample multiple species within an environment and assess several genotoxicity markers concurrently. However, DNA structure and multiple molecular, cellular and evolutionary processes are conserved across species. Therefore, results obtained from one or a few species, along with information in databases, can be used, albeit with caution, to infer the impacts of genotoxic pollution on a broader range of species and its consequences for ecosystems (Theodorakis, 2001). For this approach to be valid, the selection of an appropriate sentinel organism is crucial. It is recommended to choose species that bioaccumulate the pollutant of interest. For instance, aquatic species or small organisms are effective sentinel species for studying the toxic effects of metals, as they tend to have high abundance, wide distribution, and play key roles in complex food webs (Mussali-Galante et al., 2014).

Differentiating cellular, physiological or natural ecosystem alterations from those associated with genotoxins is not an easy task, especially in complex landscapes. Genotoxic pollution often consists of chemical mixtures, where the effects of a single genotoxin may be intensified or reduced through interactions with other chemicals. Even species adapted to exposure to a specific contaminant may experience reduced fitness and increased sensitivity when exposed to other pollutants (Brown et al., 2009). Furthermore, exposure to genotoxins can occur at low doses, but chronically. These factors pose a challenge for risk assessment in genetic ecotoxicology (Theodorakis, 2001; Mussali-Galante et al., 2014). Adding complexity to this challenge, it is important to highlight that a particular genotoxin can exert different effects on species and also may act on multiple genes and have more than one mechanism of genotoxicity. Therefore, genomics has become necessary to overcome these complexities of gene-chemical interactions.

“Ecotoxicogenomic” strategies that evaluate the effect of (I) a single chemical on multiple genes, (II) multiple chemicals on a single or few genes, (III) or multiple chemicals on multiple genes may help address the complex impacts of chemicals on the genome (Mussali-Galante et al., 2014). For example, using a toxicogenomic approach, we recently demonstrated that persistent organic pollutants impact thousands of genes across various species, adversely affecting critical biological pathways such as immune function, reproduction, and metabolism. These disruptions may contribute to both biodiversity loss and the spread of infectious diseases in varied species (Ziliotto et al., 2024a). Using the C-C chemokine receptor type 5 (CCR5) as a study model, we also recently showed that toxicogenomics can be employed to elucidate mechanisms that link exposure to environmental pollutants with increased inflammation and related consequences for multiple organisms (Ellwanger and Chies, 2024). In brief, these examples demonstrate that ecotoxicogenomic strategies indeed are useful in the study of genotoxic syndrome.

## Genotoxic syndrome and conservation

For nature conservation purposes (not considering safety/toxicology evaluations), regardless of dose, knowing if a chemical or mixture can damage the DNA is crucial. This information alone should be enough to send “warning signals” about the pollutant. In the real world, genotoxins reach the environment in varying doses, depending on the anthropogenic activity associated with biotic and abiotic factors, which can change day by day. These pollutants can be degraded or bioaccumulated in the environment, depending on their chemical composition and ecological factors. Even low exposure can potentially be harmful to populations and ecosystems since, considering genotoxic pollution, several and diverse animals will be chronically exposed to pollutants. 

Knowing that a certain pollutant has genotoxic activity should already be enough to trigger pollution control actions. This knowledge is vital for preventing and mitigating environmental damage, which is the main reason for studying genotoxic syndrome in ecology (Mussali-Galante et al., 2014). We can take an example from the field of infectious disease ecology. When analyzing the impact of metals in the environment on the spread of pathogens in animal populations, it may be more crucial to determine which general levels (whether “high” or “low” compared to “expected” levels) of specific metals or mixtures facilitate disease, rather than identifying the exact exposure doses associated with the disease (Kosoy and Biggins, 2022), which can be determined in the laboratory, but will rarely be observed in the “real world”. This strategy, which focuses on general trends rather than specific exposure doses, may overestimate risks but it is consistent with the precautionary principle (Kriebel et al., 2001). 

Besides, conservation and evolutionary issues associated with genotoxic pollutants must be interpreted in a broader context, considering the interference of other anthropogenic and natural factors. In addition to the problems related to genotoxicity, chemical pollution also has non-genotoxic toxic effects that can cause reductions in the abundance and diversity of species, influencing micro-evolution. Beyond selection, genetic drift, gene flow (e.g., due migration), recombination, and inbreeding are some additional factors that affect the resistance and susceptibility of natural populations to chemical pollutants (Brown et al., 2009; Mussali-Galante et al., 2014). For example, a decline in population size can lead to increased genetic drift and inbreeding, both phenomena often intertwined with anthropogenic activity. Inbreeding in wild populations can result in the loss of heterozygote superiority, leading to deleterious effects and reduced fitness. As part of a dangerous cycle, in addition to being linked to impaired immunocompetence and a higher incidence of congenital defects, inbreeding also increases the susceptibility of these populations to environmental stressors, including pollutants (Brown *et al.*, 2009). To enhance the resilience of animal populations, particularly those impacted by human activities and in decline, it is essential to incorporate measures that reduce genotoxic pollution into conservation strategies.

## Conclusion and future directions

Genotoxic syndrome can be summarized as a common outcome of a range of metabolic, cellular, and molecular effects induced by genotoxic pollutants across various species. The consequences of this syndrome include impaired species well-being, increased disease prevalence, and disruptions in reproduction and survival, among others. Although the concept of genotoxic syndrome has been recognized since the 1990s, it remains underexplored, particularly in terms of its broader implications beyond cancer and other aging-related conditions. Given the escalating environmental challenges globally, it is crucial to revisit and emphasize the concept of genotoxic syndrome into environmental sciences, as it offers valuable insights into how pollution contributes to biodiversity loss.

Currently, chemical pollution is intense and widespread, with rapid dissemination (Sigmund et al., 2023). Therefore, it can affect species that are evolutionarily unprepared to deal with such toxic insults, as they evolved in environments free of these anthropogenic interferences (Depledge, 1998). Similarly, the emergence of new classes of genotoxic pollutants (e.g., microplastics, complex mixtures) can cause deleterious effects on biodiversity due to the low capacity of organisms to deal with these new classes of xenobiotics. It is therefore crucial to integrate the mitigation of genotoxic pollution into broader biodiversity protection strategies, alongside efforts to conserve habitats, combat deforestation and hunting, and control climate change, among other measures. In this regard, it is imperative for governments to implement policies aimed at controlling chemical pollution at both local and regional levels, as these measures are essential for mitigating global impacts. 

Specifically, limiting the release of metals, organic pollutants, domestic and industrial wastewater, pesticides, microplastics, drug residues, radioactive material, nanomaterials, and air contaminants into the environment is essential for preserving environmental quality and safeguarding animal health from the harmful effects of genotoxic pollution. This should be done mainly through the development and application of appropriate environmental legislation, based on scientific parameters from the fields of animal health, genetics and ecotoxicology, among other disciplines. Restricting or banning the use of chemical agents with high genotoxic activity (e.g., radioactive waste, some classes of pesticides) should be considered, taking into account the specificities, needs, and legislation of each country. Industrial sector must develop technologies to limit the production and release of genotoxic pollutants through the optimization of industrial processes, recycling of chemical products, application of strict waste control strategies, and the use of chemical agent filters, among other initiatives.

Adequate environmental sanitation infrastructure and solid waste treatment systems are also essential for promoting environmental health, safeguarding organisms from multiple classes of genotoxins. Protecting ecologically sensitive areas (e.g., where high biodiversity or endangered species are observed), along with mapping and restoring regions contaminated by industrial waste, mining products, and other genotoxic pollutants, are essential strategies. Moreover, these efforts must be accompanied by environmental monitoring that includes genotoxicity indicators in animals inhabiting natural environments, as well as environmental samples (i.e., soil, air, water). Knowing which genotoxic pollutants are present in a specific site, and in what quantities, is essential for planning effective strategies to improve environmental quality.

Furthermore, individuals can play a crucial role by actively monitoring governmental actions and the activities of environmental protection agencies, while also advocating for industries to adopt sustainable production practices that minimize pollution. Reducing the consumption and demand for products associated with chemical pollutants, including genotoxic substances, is critical for mitigating their environmental impact. To achieve these objectives, it is essential to expand environmental education programs in schools and communities, with information on pollution and aspects related to genotoxicity, focusing especially on groups of individuals who handle genotoxic agents, such as farmers, electronic material (e-waste) recyclers, workers in the chemical, textile and metallurgical industries, and professionals from hospitals, laboratories and other institutions with high polluting potential.

Recent articles from our research group (Ellwanger and Chies, 2023; Ziliotto et al., 2023; 2024b; Ellwanger *et al.*, 2025a; 2025c) and other authors (Fuller et al., 2022; Tavella et al., 2024; Buralli and Connerton, 2025; Picinini-Zambelli et al., 2025) discuss in more detail some of the actions mentioned above, among others, aimed at protecting the environment from various classes of pollutants. We highly recommend these references to the reader interested in detailed information on pollution-related health issues and the prevention and mitigation of environmental pollution.

Collectively, such pollution control strategies may help mitigate the effects of genotoxic pollutants on biodiversity and ecosystems, while also safeguarding human health. However, researchers assessing biomarkers of genotoxicity in species inhabiting natural environments must place greater emphasis on the relevance of these biomarkers for wildlife, without necessarily an interpretation focused solely on human health.

## Data Availability

No new data was created in this work.
